# How many and which physicians? A comparative study of the evolution of the supply of physicians and specialist training in Brazil and Spain

**DOI:** 10.1186/s12960-020-00472-0

**Published:** 2020-04-21

**Authors:** Mário César Scheffer, Maria Pastor-Valero, Alex Jones Flores Cassenote, Antonio F. Compañ Rosique

**Affiliations:** 1 Departamento de Medicina Preventiva, Faculdade de Medicina, Universidade de São Paulo, São Paulo, Brazil; 2 Departamento de Salud Pública, Historia de la Ciencia y Ginecología, Universidad Miguel Hernández, Alicante, Spain; 3 Centro de Investigación Biomédica en Red Epidemiología y Salud Publica (CIBERESP), Madrid, Spain; 4 Departamento de Gastroenterologia, Faculdade de Medicina, Universidade de São Pauloa, São Paulo, Brazil; 5 Departamento de Patología y Cirugía, Universidad Miguel Hernández, Alicante, Spain

**Keywords:** Healthcare workforce, Physician workforce, Physician supply, Residency training

## Abstract

**Background:**

In the face of the medical workforce shortage, several countries have promoted the opening of medical schools and the expansion of undergraduate and specialization education in medicine. Few studies have compared the characteristics and effects of expanding the supply of general practitioners and specialist physicians between countries. Brazil and Spain, two countries with distinct historical processes and socioeconomic scenarios, yet both with universal public health systems and common aspects in training and medical work, have registered a significant increase in the number of physicians and can be used to understand the challenges of strategic planning for the medical workforce.

**Methods:**

This study provides a descriptive approach using longitudinal data from official databases in Brazil and Spain from 1998 to 2017. Among the comparable indicators, the absolute numbers of physicians, the population size, and the physician’s ratio by inhabitants were used. The number of medical schools and undergraduate places in public and private institutions, the supply of residency training posts, and the number of medical specialists and medical residents per 100 000 inhabitants were also used to compare both countries. Seventeen medical specialties with the highest number of specialists and comparability between the two countries were selected for further comparison.

**Results:**

Due to the opening of medical schools, the density of physicians per 1 000 inhabitants grew by 28% in Spain and 51% in Brazil between 1998 and 2017. In that period, Spain and Brazil increased the supply of annual undergraduate places by 60% and 137%, respectively. There is a predominance of private institutions providing available undergraduate places, and the supply of medical residency posts is smaller than the contingent of medical graduates/general practitioners each year.

**Conclusion:**

Both countries have similar specialist densities in cardiology, dermatology, and neurosurgery specialties. However, family medicine and community in Spain has 91.27 specialists per 100 000 inhabitants, while in Brazil, the density is only 2.64. The comparative study indicated the complexity of the countries’ decisions on increasing the medical supply of general practitioners and specialist physicians. Research and planning policies on the medical workforce must be aligned with the actual health needs of populations and health systems.

## Introduction

In order to better meet the health needs of human populations, health workforce planning should consider changes in health systems and countries’ epidemiological contexts and new demands related to aging and life expectancy [1–3]. In this sense, the coordination of national databases on health professionals and improvement of evidence on human resource policies is recommended [4].

The shortage of physician workforce has been reported worldwide as a serious problem in recent decades, despite the recent increase in their production worldwide [5, 6]. Several strategies such as opening medical schools, expanding graduation and specialization education, and changing migration policies and strategic policies affecting retirement and retention of physicians in health services have been used by governments and legislators to change the supply of physicians [7].

The overall increase in the number of available physicians was marked by particular trends in medicine such as the increased participation of women and the presence of more specialists than general practitioners [8]. Nevertheless, marked inequalities still persist regarding the supply of specialists, physicians’ geographical distribution (urban, suburban versus peripheral, and rural regions), and distribution across public and private sectors and across the different levels of health services (primary, secondary, and tertiary care) [9].

Thus, it is crucial to promote evidence-based analyses targeting the nature and effects of the growth of physician workforce, especially considering that comparative studies are relevant to better comprehend complex but similar issues arising from different national health systems [10]. The present study aims to compare the evolution of the physician supply and specialist training from 1998 to 2017 in Spain and Brazil, two countries with distinct socioeconomic and demographic characteristics, yet both with universal health care systems.

## Methods

This descriptive study uses longitudinal data from official public databases from Brazilian and Spanish governments and medical institutions. The decision to compare both countries was based on the following: (I) access to a database of physicians and recent medical demography reports for both countries [11, 12]; (II) the similarities between the National Health System (SNS) of Spain and the Unified Health System (SUS) of Brazil, both free universal public systems; (III) and the similar criteria required to obtain medical certification (6 years of medical training) and specialization (medical residency training) in both countries.

The total number of physicians, population size, and density per inhabitant between 1998 and 2017 were determined and used as comparable indicators. The analysis was based on a definition of licensed physicians that considers all professionals registered in an official national corporative agency with the right to legally practice the profession in the country [13].

To describe the evolution of the supply of physicians, the number of public and private schools was used, as well as the total number of medical students in training per 100 000 inhabitants.

In order to determine the supply of specialists, the density of specialists and physicians undergoing residency/internship programs per 100 000 inhabitants (public programs of medical residency in Spain and Brazil) was considered.

From the 52 medical specialties recognized in Spain [12] and the 54 specialties recognized in Brazil [14], 17 specialties with the highest number of specialists, nomenclature compatibility, and similar training content in both countries were selected for comparison.

The growth rates of the population and the number of physicians were calculated based on the previous year with the following equation: ((*b* − *a*)/*a*) × 100, where *a* represents the number of individuals at the end of the period considered and *b* the number of individuals at the beginning of the period considered. The accumulated growth rate calculation considered the complete observational period (from 2001 to 2017). The calculation of physician’s density has considered the following equation: ((*x*/*y*) × 1000 inhabitants), where *x* refers to the number of physicians and *y* to the population size in the respective year analyzed.

This research was approved by the Research Ethics Committee of the Faculty of Medicine of the University of São Paulo (CEP number 797.424).

## Results

**Fig. 1:**
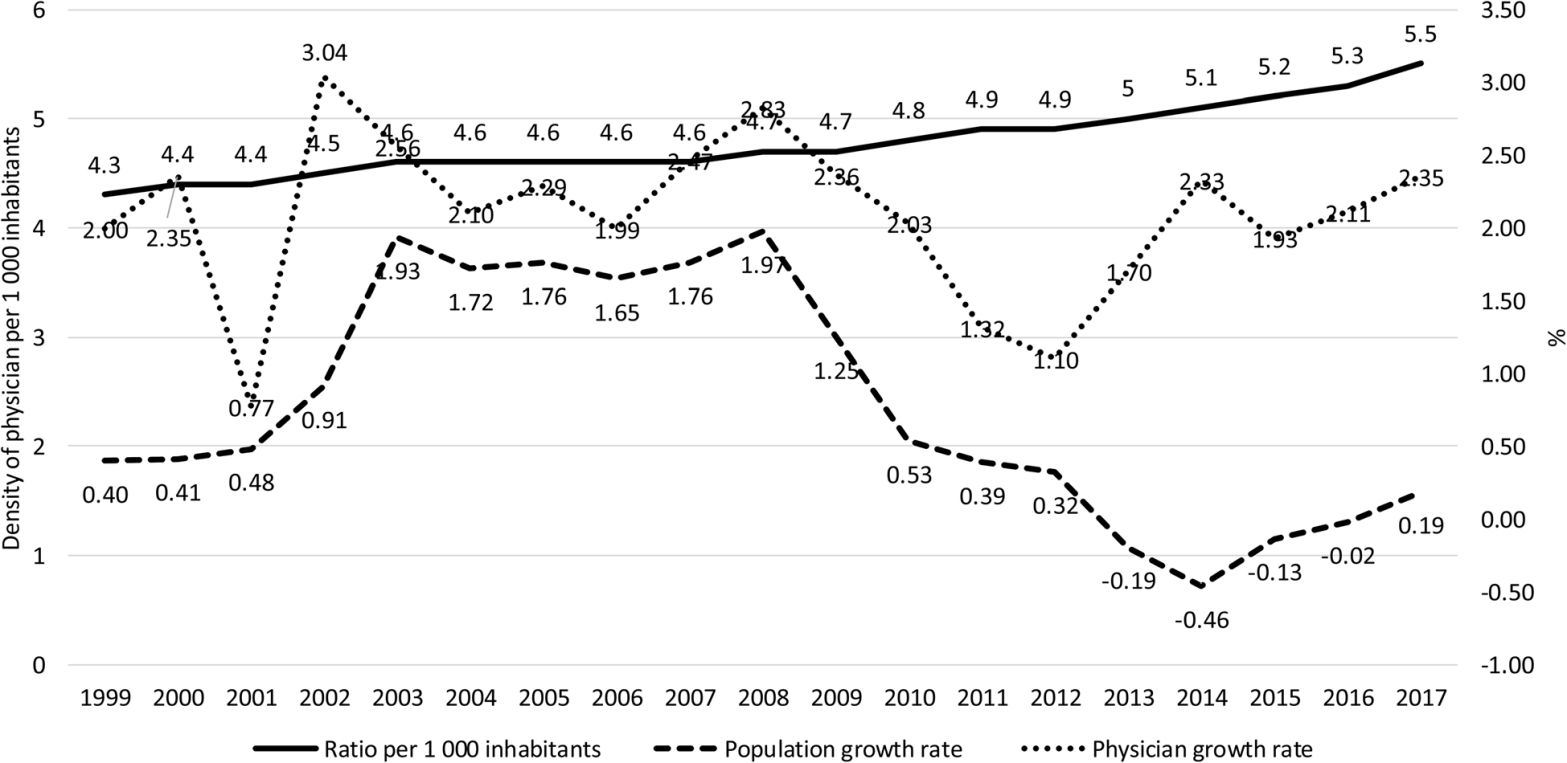
Historical evolution of physician growth rate, population growth rate, and density of physicians per 1 000 inhabitants in Spain, from 1999 to 2017

**Fig. 2:**
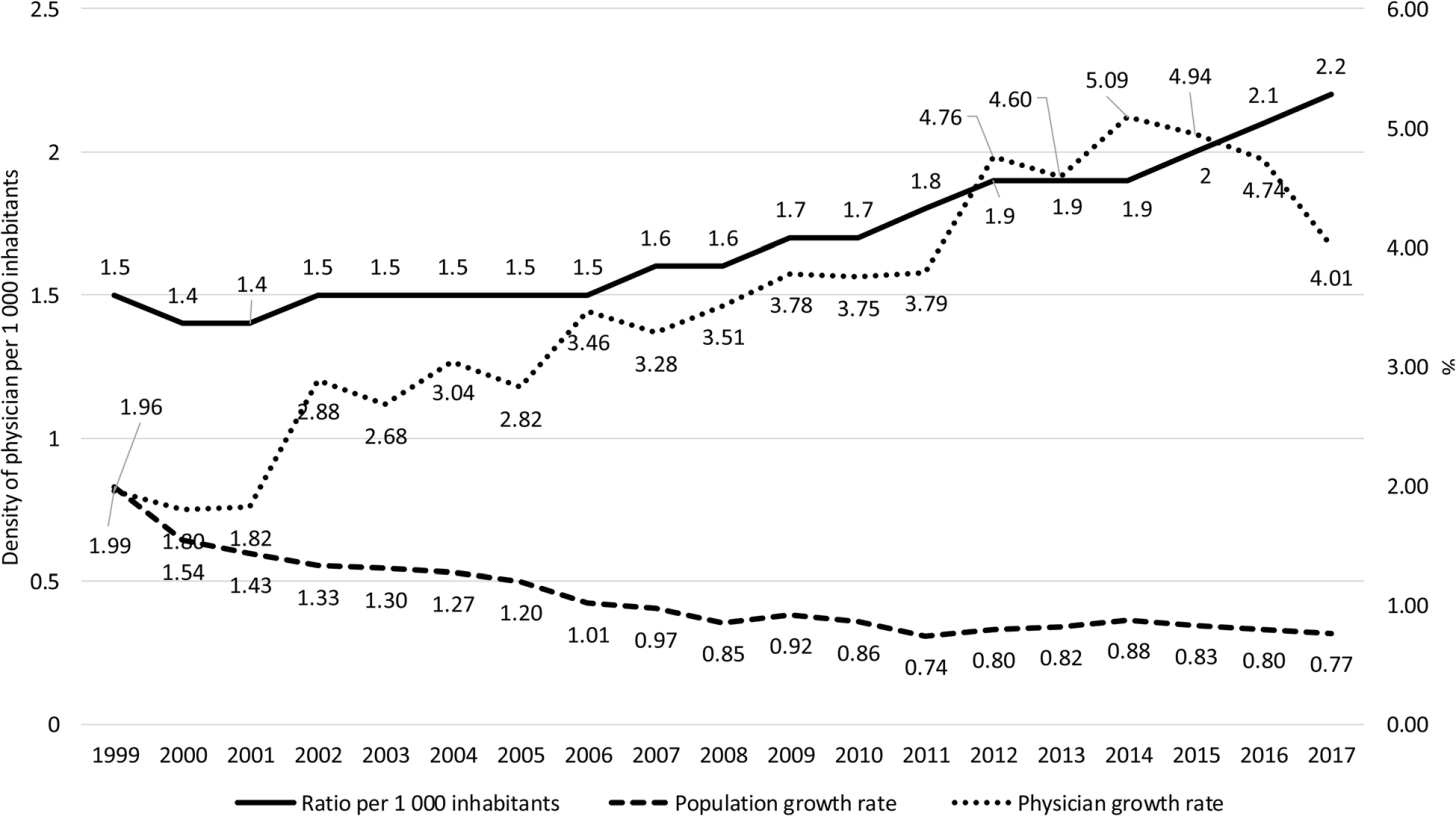
Historical evolution of physician growth rate, population growth rate, and density of physicians per 1 000 inhabitants in Brazil, from 1999 to 2017

Between 1998 and 2017, the density of physicians per 1 000 inhabitants grew by 27.6% in Spain and 51.1% in Brazil. In the same period, it was also observed that the population of physicians has grown faster than the general population in both countries (Figs. 1 and 2). In Spain, the number of physicians increased by 48% in the two decades—from 171 494 to 253 796—while in Brazil, it has increased 94%—from 234 685 to 451 777 (Supplementary table 1).

From 2001 to 2017, residency training posts in Spain increased from 4 371 per year (in 29 medical schools) to 6 995 (in 44 schools), a 60% increase in total posts available. In the same period, residency training posts in Brazil increased from 13 173 (in 109 schools) to 30 971 (in 289 schools), an increase of 136.8% (Table 1).

**Table 1:** Historical evolution of total medical students per year and density of medical students per 100 000 inhabitants in Spain and Brazil (2001–2017)

Year	Spain		Brazil	
	Total medical students	Density^1^	Total medical students	Density^1^
2001	4 371	10.7	13 173	7.4
2002	4 359	10.6	13 732	7.7
2003	4 359	10.4	14 724	8.1
2004	4 343	10.2	16 497	9.0
2005	4 343	10.0	17 575	9.4
2006	4 726	10.7	19 250	10.2
2007	5 032	11.2	20 515	10.8
2008	5 871	12.9	21 279	11.1
2009	6 229	13.5	21 379	11.1
2010	6 673	14.3	21 649	11.1
2011	6 949	14.9	21 987	11.2
2012	7 061	15.1	23 985	12.1
2013	6 977	14.9	24 587	12.2
2014	6 905	14.8	27 950	13.8
2015	6 847	14.7	29 376	14.4
2016	6 877	14.8	30 002	14.6
2017	6 995	15.0	30 971	14.9

Sources: Spain: Instituto Nacional de Estadística (INE), Organización Médica Colegial de España (OMC). Brazil: Instituto Brasileiro de Geografia e Estatística (IBGE), Ministério da Educação (MEC)

^1^Total of medical students/year per 100 000 population

In both countries, the increase in physicians’ number was accompanied by the expansion of private medical schools. In Spain, from 2001 to 2017, private undergraduate places increased from 230 to 1 335, which represented an accumulated growth of 480%; of the total places in Spain in 2017 (6 995), 19.1% of them (1 335) were provided by private schools. In Brazil, in the same period, private undergraduate places increased from 7 107 to 21 766, resulting in an accumulated growth of 206.2%; of the total places in Brazil in 2017 (29 271), 70.3% of them (21 766) were private.

Spain and Brazil had respectively 10.7 and 7.4 medical residency posts/100 000 inhabitants in 2001, a density that increased to 14.9 in Brazil and 15.0 in Spain in 2017. The rate of posts projects a continuous increase in the number of physicians in both countries. However, in 2017, 37.5% of the physicians (169 479) in Brazil [11] had no medical specialty, and this was a similar situation for 30.5% of the physicians (77 369) in Spain [12]. This occurred either because physicians did not want to apply for a medical residency program or they were not admitted to a residency program.

The number of residency posts has not been sufficient to match the number of recently graduated physicians. Spain registered 6 995 medical graduates in 2017 and had 6 515 medical residency posts in 2018. In Brazil, on the other hand, there were 30 971 recently graduated physicians in 2017, with only 19 314 medical residency posts in 2018.

In both countries, residency posts in a given year are allocated competitively between the following three groups: (1) by newly trained physicians who graduated in the previous year, (2) by physicians who are already specialists but are interested in obtaining another specialty, and (3) by the large contingent of physicians without a specialty accumulated over time. In the case of Spain, some residency posts are filled by foreigners (in 2018, they occupied 9.2% of the posts).

Table 2 shows the distribution of specialist physicians in the 17 selected medical specialties and medical residents per 100 000 inhabitants in both countries. The most common specialty in Spain is family medicine and community, with 91.27 specialists and 14.39 medical residents per 100 000 inhabitants, while in Brazil, these densities are much lower: 2.64 and 0.75, respectively. In Brazil, on the other hand, the most frequent specialty is internal medicine/clinics, with 20.58 specialists and 2.1 medical residents per 100 000 inhabitants, while in Spain, the densities are 17.2 and 3.3, respectively. The most similar specialties between the two countries considering density are the following: cardiology (7.6 specialists and 1.6 residents in Spain, 7.4 and 0.5 in Brazil), dermatology (4.5 and 0.6 in Spain, 4.0 and 0.3 in Brazil), and neurosurgery (1.8 and 0.3 in Spain, 1.5 and 0.2 in Brazil).

**Table 2:** Distribution of medical specialists and medical resident students according to selected specialties in Spain and Brazil, 2017

Specialties	Spain						Brazil					
	Medical specialists^1^	Percentage	*D*^2^	Medical resident students^3^	Percentage^4^	*D*^5^	Medical specialists^1^	Percentage	*D*^2^	Medical resident students^3^	Percentage^4^	*D*^5^
Family medicine and community	42 465	24.1	91.3	6 693	24.4	14.4	5 486	1.4	2.6	1 554	4.4	0.7
Pediatrics	11 388	6.5	24.5	1 612	5.9	3.5	39 234	10.3	18.9	3 448	9.8	1.7
Internal medicine/clinics	8 027	4.5	17.2	1 546	5.6	3.3	42 728	11.2	20.6	4 466	12.7	2.1
Anesthesiology	7 914	4.5	17.0	1 245	4.5	2.7	23 021	6.0	11.1	2 579	7.3	1.2
Gynecology and obstetrics	7 448	4.2	16.0	984	3.6	2.1	30 415	8.0	14.6	3 018	8.6	1.4
Orthopedics and traumatology	6 289	3.6	13.5	1 123	4.1	2.4	15 598	4.1	7.5	2 292	6.5	1.1
Psychiatry	5 780	3.3	12.4	901	3.3	1.9	10 396	2.7	5.0	1 448	4.1	0.7
Radiology	5 123	2.9	11.0	843	3.1	1.8	12 233	3.2	5.9	1 290	3.7	0.6
General surgery	5 212	3.0	11.2	887	3.2	1.9	34 065	8.9	16.4	2 895	8.2	1.4
Ophthalmology	4 749	2.7	10.2	664	2.4	1.4	13 825	3.6	6.7	1 173	3.3	0.6
Cardiology	3 568	2.0	7.7	766	2.8	1.6	15 516	4.1	7.5	1 073	3.1	0.5
Neurology	2 512	1.4	5.4	486	1.8	1.0	5 104	1.3	2.5	826	2.3	0.4
Urology	2 298	1.3	4.9	469	1.7	1.0	5 328	1.4	2.6	521	1.5	0.2
Dermatology	2 135	1.2	4.6	311	1.1	0.7	8 317	2.2	4.0	647	1.8	0.3
Otolaryngology	2 628	1.5	5.6	312	1.1	0.7	6 373	1.7	3.1	588	1.7	0.3
Neurosurgery	846	0.5	1.8	175	0.6	0.4	3 298	0.9	1.6	538	1.5	0.3
Plastic surgery	1 160	0.7	2.5	174	0.6	0.4	6 304	1.7	3.0	439	1.2	0.2
Other specialties^6^	56 884	32.2	122.3	8 211	30.2	17.6	10 4265	27.3	50.2	6 383	18.1	3.1
Total	176 426	100.0	379.2	27 402	100.0	58.9	381 506	100.0	183.7	35 178	100.0	16.94

Sources: Spain : Instituto Nacional de Estadística (INE), Organización Médica Colegial de España (OMC), Ministerio de Sanidad, Servicios Sociales e Igualdad; Brazil: Instituto Brasileiro de Geografia e Estatística (IBGE), Ministério da Educação (MEC). Conselho Federal de Medicina (CFM)

^1^Number of medical specialists registered in 2017

^2^Density of medical specialists per 100 000 inhabitants

^3^Number of medical resident students per specialty in 2017

^4^Percentage of medical resident students over the total of medical residency posts for a given specialty

^5^Density of resident students per 100 000 inhabitants

^6^Others: 35 specialties out of 52 in Spain and 37 out of 54 specialties in Brazil

## Discussion

Based on the shortage of the medical workforce and specialists and poor availability of public health services in Spain [15–17] and Brazil [18], both countries have followed, in the last 20 years, the international tendency [7] to increase the number of physicians in a greater proportion than the population growth. The most significant growth occurred in Brazil, although in 2017, the country had less than half of the density of physicians found in Spain, mostly reflecting the late Brazilian demographic transition.

The opening of new medical schools and residency training posts has generated a continuous increase in the medical workforce in both countries, in spite of the losses due to retirement, death, and migration flows to other countries (in the case of Spain). Yet, there is no evidence that the opening of a large number of medical schools in Brazil in such a short period of time has been preceded by appropriate and evidence-based planning, and the expansion observed is probably a consequence of the implementation of recent policies that induced the opening of new medical schools, such as the More Doctors Law, enacted in 2013 [19].

In both Spain and Brazil, the increase in the number of physicians is associated with the opening of new medical schools, mostly private. Previous studies regarding the privatization of medical education, a global phenomenon [20, 21], have pointed out that there are different degrees of governmental regulation to assure the quality of private and public medical courses [22]. The possibility to pay, instead of true academic merit, usually prevails in the admission process of most private schools [23]; the students’ performance in private schools is considered to be lower than those from public schools [24]; and the private medical education market is mostly driven by multinational private educational groups [24]. Private medical education in Brazil registered lower indicators of quality and performance than public education, and the high costs of private courses favor the access of students of better socioeconomic status [24]. In Spain, the increase in the number of graduates from private medical schools may lead to the possible erosion of meritocracy and equity in access to the medical profession [25]. The quality of teaching in Spanish private medical schools is very varied. A particularly problematic aspect is to know what the admission criteria are in these private institutions. In the public schools, there is only one criterion that is the prioritization by the “selectivity” grade: a state examination. Only the best students can be admitted. However, in most of the private schools, the criteria are different. In many of them, this note of “selectivity” is not taken into account and a great importance is given to personal interviews and, therefore, completely subjective, allowing inequalities.

The number of medical schools has increased worldwide, but unequal geographical distribution still prevails. Moreover, medical schools may lack qualified professor infrastructure, a qualified clinical practice environment, and appropriate conditions to provide adequate specialist medical training [26]. In Spain, previous reports had shown that there was an insufficient number of medical professors and a saturation of students in hospitals and health centers [27]; this was mostly attributed to the opening of new medical schools.

In both countries, every year, the number of newly graduated physicians is greater than the number of medical residency training posts. In Brazil [28] and Spain [29], medical residency training is considered to be the best model for training specialists. The consequence is the development of a “pool,” a growing contingent of physicians without a specialty, that already accounts for 30% and 37% of the medical workforce in Spain and Brazil, respectively. In Spain, there is an aggravating factor because a medical specialty is required in order to work in the public health system; this does not occur in Brazil.

By comparing the density of specialists per 100 000 inhabitants between Brazil and Spain, it is emphasized that there are two distinct groups. The first one includes specialties with greatest differences of density between both countries, such as family medicine and community, psychiatry, orthopedics and traumatology, pediatrics, internal medicine, radiology, anesthesiology, urology, neurology, general surgery, ophthalmology, and otorhinolaryngology. The second group is made up of specialties with no significant differences in density between both countries, such as cardiology, dermatology, neurosurgery, gynecology and obstetrics, and plastic surgery.

The great divergence in density found in the family medicine and community specialty (in Spain, 91.27, and in Brazil, only 2.64 specialists per 100 000 inhabitants) may be explained by the different qualification requirements for physicians working in primary care in both countries. On the other hand, psychiatrists and orthopedists, for example, have a lower density in Brazil. This is in contrast to the high prevalence of mental disorders and injuries due to external causes that occur in Brazil [30].

Such disparities or similarities between Brazil and Spain do not seem to express the sociodemographic characteristics and health needs of their populations or even common epidemiological challenges such as chronic non-transmissible diseases as the main cause of morbidity and mortality in both countries [30, 31].

As for the medical residency, besides the insufficient supply, the distribution of physicians undergoing residency training across the different specialties is highly similar to the distribution of the specialists in the actual job market. For example, in Spain, 4.5% of the specialists are anesthesiologists, while 4.5% of the residents’ population also take the same specialty. In Brazil, gynecologists and obstetricians are 8.0% of specialists and 8.6% of residents. Yet, the percentage of residents in training is higher than the percentage of active specialists in some specialties, such as family medicine and community, orthopedics and traumatology, and psychiatry. This reflects the impact of recent policies to induce more medical residency training posts in these specialties, which may increase the future supply of specialists in these areas.

The perpetuation of the same number of medical residency posts over time found in several specialties can be attributed in part to the lack of planning to modulate the supply of training posts to health needs and demands, but may also be related to the corporatism of institutions and programs and teachers that oppose flexibility (increase or decrease) in the supply of posts. In addition, the increased participation of the private sector in health systems in Brazil and Spain has led to the maintenance of large numbers of physicians dedicated to specialized care and less expansion of the training of physicians required for primary care.

Considering that the decisions on the supply of undergraduate training posts and specialization in medicine should be part of the same planning, Pérez and López-Valcárcel [25] propose that, given the shortage of specialists projected for Spain, the availability of medical students in training should gradually decrease while increasing the posts for medical residency in strategic specialties.

Although in some specialties slight variations in the proportion between specialists and residents in both countries are found, the results may indicate an absence of planning or outdated planning for medical training in the specialties. The distribution of physicians in specialties should consider the following: deficiencies in the health system, the main determinants of health and disease, and population aging and its multiple morbidities. Such conditions would require more specialists in internal medicine/clinics, primary care, geriatrics, and other specialties which are more likely to diagnose and treat health conditions that become increasingly frequent in a timely fashion [9, 32, 33].

Thus, the present study corroborates the global literature on countries’ responses to overcome the shortage of the medical workforce by demonstrating that the opening of medical schools cannot be a fully effective response [34]. Planned policies to address inequalities in regional distribution and between specialties are needed [35] and should consider demographic, educational, and labor market variables [36]. Such policies should also be based on the real demands of health services in order to provide care for an aging population with continuously growing morbidity from chronic diseases [37]. Also, they should promote the application of innovative solutions, interdisciplinary work, and better use of technology to increase the effectiveness and efficiency of health care [38].

The strength of this study was to compare two countries with similarities in their health systems and regulation of the medical profession. It relied on national databases and a comparable series of historical data. But there are also limitations. The analysis only used data up to 2017 and therefore did not capture recent phenomena. For example, in 2013, Brazil approved the More Doctors Law [19], which expands medical courses and medical residency programs, and this will impact the future configuration of the medical workforce. Also, the study did not cover several levels of inequalities such as geographic and populational differences, social and economic development, health system financing, and health indicators that separate Brazil from Spain. All of these aspects can influence the supply and distribution of professionals and access of the population to medical care.

## Conclusions

The comparative case of Brazil and Spain indicates the complexity that surrounds the decisions of countries to supply general and specialist physicians. This study shows that in both countries, specialized training programs are insufficient to meet the demand generated by the opening of new medical schools. It also shows that there is an inadequate distribution of specialists among medical specialties and that the expansion of medical education is mainly driven by the opening of private educational institutions. Such findings need to be considered in human resources planning and assessment policies for the health systems in Brazil and Spain. Decisions on the provision of new physicians need to be accompanied by the characterization of possible geographical imbalances, the quality of training, the inequalities in the access to health care, and the capacity to respond to people’s demands and health service needs.

The present study provides evidence that the implementation of policies aimed at increasing the number of general practitioners and specialists should be preceded by adequate planning that considers the dynamics of the medical labor market, health system characteristics, regional and local inequalities, and novel epidemiological scenarios, including the demands related to aging and increased life expectancy. To meet these goals, it is highly recommended to coordinate national databases and produce evidence on medical workforce and the health system, which includes the development of dynamic prediction models that use variables of both the supply and demand of physicians.

## Supplementary information

**Additional file 1.** Distribution of physicians, population, and physicians per 1000 inhabitants in Spain and Brazil, 1998–2017.

## Data Availability

The datasets generated and/or analyzed during the current study are not publicly available due to ethical issues related to participant confidentiality imposed by the Ethics Committee of the Medical School of the University of São Paulo. Data from this paper are available upon request to the Ethics Committee of the Medical School of the University of São Paulo. Mailing address: 251 Dr. Arnaldo Avenue, Cerqueira César, São Paulo, SP 01246-000, Brazil. Phone: + 55 (11) 3893–4401: Dr. Maria Aparecida Azevedo Koike Folgueira.

## Abbreviations

SNS: National Health System of Spain
SUS: Unified Health System
MIR: Internal resident physician
RM: Medical residency
