# Supplementary material for: How many and which physicians? A comparative study of the evolution of the supply of physicians and specialist training in Brazil and Spain
Source: Hum Resour Health. 2020 Apr 21;18:30. doi: 10.1186/s12960-020-00472-0 (PMC7171868; doi:10.1186/s12960-020-00472-0)
Supplement: Supplementary file 1 — Additional file 1. Distribution of physicians, population, and physicians per 1000 inhabitants in Spain and Brazil, 1998–2017. [file 12960_2020_472_MOESM1_ESM.docx]

**Additional file**

**How many and which physicians? A comparative study of the evolution of the supply of physicians and specialist training in Brazil and Spain**

**Supplementary table 1.** Distribution of physicians, population, and physicians per 1,000 inhabitants in Spain and Brazil, 1998-2017

|  | **SPAIN** | | | | **BRAZIL** | | | |
| --- | --- | --- | --- | --- | --- | --- | --- | --- |
| **Year** | **Population^1^** | **Physicians** | **Density^2^** | **Numeric growth^3^** | **Population^1^** | **Physicians** | **Density^2^** | **Numeric growth^3^** |
| 1998 | 40,143,449 | 171,494 | 4.27 | - | 168,490,311 | 234,685 | 1.39 | - |
| 1999 | 40,303,568 | 174,916 | 4.34 | 3,422 | 171,847,554 | 239,282 | 1.39 | 4,597 |
| 2000 | 40,470,182 | 179,033 | 4.42 | 4,117 | 174,490,693 | 243,581 | 1.40 | 4,299 |
| 2001 | 40,665,545 | 180,417 | 4.44 | 1,384 | 176,985,826 | 248,019 | 1.40 | 4,438 |
| 2002 | 41,035,271 | 185,908 | 4.53 | 5,491 | 179,332,960 | 255,174 | 1.42 | 7,155 |
| 2003 | 41,827,836 | 190,665 | 4.56 | 4,757 | 181,671,437 | 262,013 | 1.44 | 6,839 |
| 2004 | 42,547,454 | 194,668 | 4.58 | 4,003 | 183,981,024 | 269,986 | 1.47 | 7,973 |
| 2005 | 43,296,335 | 199,123 | 4.60 | 4,455 | 186,184,264 | 277,606 | 1.49 | 7,620 |
| 2006 | 44,009,969 | 203,091 | 4.61 | 3,968 | 188,070,562 | 287,219 | 1.53 | 9,613 |
| 2007 | 44,784,659 | 208,098 | 4.65 | 5,007 | 189,891,688 | 296,651 | 1.56 | 9,432 |
| 2008 | 45,668,938 | 213,977 | 4.69 | 5,879 | 191,512,814 | 307,062 | 1.60 | 10,411 |
| 2009 | 46,239,271 | 219,031 | 4.74 | 5,054 | 193,272,754 | 318,663 | 1.65 | 11,601 |
| 2010 | 46,486,621 | 223,484 | 4.81 | 4,453 | 194,932,694 | 330,614 | 1.70 | 11,951 |
| 2011 | 46,667,175 | 226,424 | 4.85 | 2,940 | 196,376,496 | 343,153 | 1.75 | 12,539 |
| 2012 | 46,818,216 | 228,917 | 4.89 | 2,493 | 198,746,886 | 359,492 | 1.81 | 16,339 |
| 2013 | 46,727,890 | 232,816 | 4.98 | 3,899 | 201,002,714 | 376,020 | 1.87 | 16,528 |
| 2014 | 46,512,199 | 238,240 | 5.12 | 5,424 | 202,768,562 | 395,167 | 1.95 | 19,147 |
| 2015 | 46,449,565 | 242,840 | 5.23 | 4,600 | 204,450,649 | 414,707 | 2.03 | 19,540 |
| 2016 | 46,440,099 | 247,958 | 5.34 | 5,118 | 206,081,432 | 434,374 | 2.11 | 19,667 |
| 2017 | 46,527,039 | 253,796 | 5.45 | 5,838 | 207,660,929 | 451,777 | 2.18 | 17,403 |

*Sources: SPAIN : Instituto Nacional de* Estadística (INE) *e Organización Médica Colegial de España (OMC); BRAZIL: Instituto Brasileiro de Geografia e Estatística (IBGE) e Conselho Federal de Medicina (CFM)*

*^1^ Population*

*^2^ Physicians per 1,000 inhabitants*

*^3^ Growth of number of physicians compared to previous year*
